# Statistics of Tuberculosis in Children

**Published:** 1914-01

**Authors:** 


STATISTICS OF TUBERCULOSIS IN CHILDREN. Jules Comby of Paris in the Section of Pædiatrics of the International Congress of Medicine, reported 638 cases of tuberculosis in 1,675 necropsies—38%—in the compined hospital statistics of Paris. In the age groups, the number of deaths ranged from 73 to 415. We give only the percentages of tuberculous infection, as the figures in the report we have seen do not agree exactly:

Up to three months, 1.82% tuberculous.
Three to six months, 18.18% tuberculous.
Six to twelve months, 26.25% tuberculous.
One to two years, 40.72% tuberculous.
Two to five years, 60.% tuberculous.
Five to ten years, 67.15% tuberculous.
Ten to fifteen years, 71.23% tuberculous.

